# Use of an Intubating Stylet as a Guide to Complete Uterine Curettage Complicated by Uterine Perforation

**DOI:** 10.1155/2013/195383

**Published:** 2013-09-01

**Authors:** Jonathan D. Baum, Douglas J. Sherlock, Andrew L. Atkinson

**Affiliations:** Department of Obstetrics and Gynecology, Jersey Shore University Medical Center, 1945 State Route 33, Neptune, NJ 07753, USA

## Abstract

Completion of uterine curettage may be challenging following uterine perforation even under sonographic and laparoscopic monitoring. This report illustrates the use of a flexible intubating stylet as a guide to place the suction curette into the uterine cavity when sonography and laparoscopy alone are not successful. Use of a malleable instrument such as an intubating stylet as a guide should be considered an option when insertion of the suction curette into the uterine cavity is complicated by anatomic variation and uterine perforation.

## 1. Introduction

The most common complication of dilation and curettage is uterine perforation with an incidence ranging from 2 to 19.8 per 1000 procedures [1–4]. Risk factors for uterine perforation include resident physician performance of the procedure, multiparity, advancing gestational age, general anesthesia, and retroversion of the uterus [2, 4]. In 1958, Word et al. wrote of the fallacy of simple uterine curettage commenting that the procedure may exhaust even the most experienced gynecologist [3]. Multiple authors have recommended completion of a difficult uterine curettage be done under ultrasound guidance, laparoscopic visualization, or both [2, 4–6].

## 2. Case

26-year-old female gravida 3 para 2 underwent suction curettage for a missed abortion at 6-7 weeks of gestation determined by transvaginal ultrasound. Past history was significant for two prior cesarean deliveries. Her body mass index was normal. Examination under anesthesia revealed a retroverted uterus about 8 weeks size with limited mobility presumably due to adhesions. The cervix was dilated, and an 8 mm curved curette was placed. Suction curettage was performed, but no products of conception were obtained.

**Figure 1:**
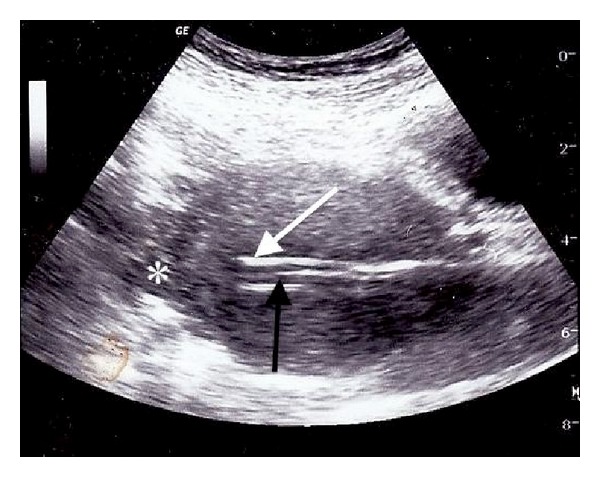
Under ultrasound guidance, the curette (white Arrow) was advanced over the stylet (black Arrow) into the uterine cavity (fundus star).

**Figure 2:**
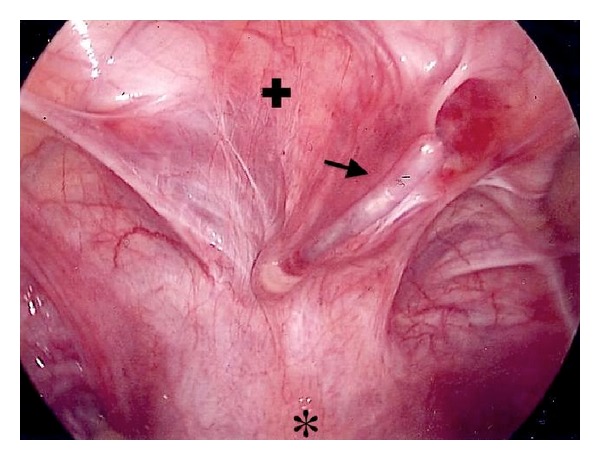
Laparoscopic view of the stylet (black Arrow) placed through the false tract below the bladder (plus symbol) and above the uterus (star).

Transabdominal ultrasound showed an intact gestational sac at the fundus; however, the suction curette was noted to be passing between the uterus and the bladder suggesting uterine perforation. Attempts to direct the suction curette away from the false tract and toward the gestational sac were not successful. A 10-French intubating stylet was shaped to approximate the degree of uterine retroversion and was directed successfully into the uterine cavity under sonographic monitoring. The suction curette was then threaded over the stylet into the uterine cavity (Figure 1). The curettage was then completed under laparoscopic visualization. The stylet was redirected into the false tract, and uterine perforation was confirmed in an area of extensive cohesive adhesions between the uterus and bladder (Figure 2). No other internal injuries were noted. The postoperative course was uncomplicated.

## 3. Discussion

While sonography was helpful in identifying the gestational sac at the fundus in this patient, it was not helpful in guiding the suction curette into the uterine cavity. While laparoscopy was helpful in confirmation of uterine perforation, it was not helpful in completion of the uterine curettage. 

Many procedures are performed in an outpatient setting which may limit the availability of an intubating stylet. Any flexible sounding device could be used in place of the stylet as described in our case. Use of a flexible curette and a malleable uterine sound shaped to match the angle of the cervical canal has been described [7]. In our case, flexible curettes are not routinely available at our institution. Our use of rigid suction curettes required the use of a flexible guide over which the curette could be passed. Traditional uterine sounds, though available, have nondetachable handles which precluded their use as a guide in our case. 

Another described method includes deferred evacuation for an additional week. This method would allow for healing and has been described in the stable patient with a small midline perforation and an intact gestational sac [7]. The concerns in regard to this method include increased risk of bleeding, infection, additional sedation, or anesthesia and may be psychologically difficult for the patient.

By combining well-known techniques with ingenuity, we were able to complete the evacuation after perforation was identified thereby avoiding the risks of deferred evacuation. 

Our case demonstrates that completion of uterine curettage may be challenging following uterine perforation even with sonographic and laparoscopic monitoring. The use of an intubating stylet should be considered to complete the procedure. When facing a difficult evacuation, the experienced practitioner should be able to complete the procedure through proper preparation, anticipation, and improvisation [7]. 

## References

[1] Gershenson DM, DeCherney AH, Curry SL (1993). *Operative Gynecology*.

[2] Rock JA, Jones HW (2008). *TeLinde's Operative Gynecology*.

[3] Word B, Gravlee LC, Wideman GL (1958). The fallacy of simple uterine curettage. *Obstetrics and Gynecology*.

[4] Kaali SG, Szigetvari IA, Bartfai GS (1989). The frequency and management of uterine perforations during first-trimester abortions. *American Journal of Obstetrics and Gynecology*.

[5] Romero R, Copel JA, Jeanty P (1985). Sonographic monitoring to guide the performance of postabortal uterine curettage. *American Journal of Obstetrics and Gynecology*.

[6] Wheeless CR (1997). *Atlas of Pelvic Surgery*.

[7] Paul M, Lichtenberg ES, Borgatta L, Grimes DA, Stubblefield PG, Creinin MD (2009). *Management of Unintended and Abnormal Pregnancy, Comprehensive Abortion Care*.

